# Comparison of metagenomic and traditional methods for diagnosis of *E. coli* enteric infections

**DOI:** 10.1128/mbio.03422-23

**Published:** 2024-03-15

**Authors:** C. Royer, N. V. Patin, K. J. Jesser, A. Peña-Gonzalez, J. K. Hatt, G. Trueba, K. Levy, K. T. Konstantinidis

**Affiliations:** 1 School of Biological Sciences, Georgia Institute of Technology, Atlanta, Georgia, USA; 2 Department of Environmental and Occupational Health, University of Washington, Seattle, Washington, USA; 3 Max Planck Tandem Group in Computational Biology, Department of Biological Sciences, Universidad de los Andes, Bogotà, Colombia; 4 School of Civil and Environmental Engineering, Georgia Institute of Technology, Atlanta, Georgia, USA; 5 Institute of Microbiology, Universidad San Francisco de Quito, Quito, Ecuador; University of Cambridge, Cambridge, United Kingdom

**Keywords:** diarrhea, diagnostics, metagenomics, *E. coli*

## Abstract

Diarrheagenic *Escherichia coli*, collectively known as DEC, is a leading cause of diarrhea, particularly in children in low- and middle-income countries. Diagnosing infections caused by different DEC pathotypes traditionally relies on the cultivation and identification of virulence genes, a resource-intensive and error-prone process. Here, we compared culture-based DEC identification with shotgun metagenomic sequencing of whole stool using 35 randomly drawn samples from a cohort of diarrhea-afflicted patients. Metagenomic sequencing detected the cultured isolates in 97% of samples, revealing, overall, reliable detection by this approach. Genome binning yielded high-quality *E. coli* metagenome-assembled genomes (MAGs) for 13 samples, and we observed that the MAG did not carry the diagnostic DEC virulence genes of the corresponding isolate in 60% of these samples. Specifically, two distinct scenarios were observed: diffusely adherent *E. coli* (DAEC) isolates without corresponding DAEC MAGs appeared to be relatively rare members of the microbiome, which was further corroborated by quantitative PCR (qPCR), and thus unlikely to represent the etiological agent in 3 of the 13 samples (~23%). In contrast, ETEC virulence genes were located on plasmids and largely escaped binning in associated MAGs despite being prevalent in the sample (5/13 samples or ~38%), revealing limitations of the metagenomic approach. These results provide important insights for diagnosing DEC infections and demonstrate how metagenomic methods can complement isolation efforts and PCR for pathogen identification and population abundance.

**IMPORTANCE:**

Diagnosing enteric infections based on traditional methods involving isolation and PCR can be erroneous due to isolation and other biases, e.g., the most abundant pathogen may not be recovered on isolation media. By employing shotgun metagenomics together with traditional methods on the same stool samples, we show that mixed infections caused by multiple pathogens are much more frequent than traditional methods indicate in the case of acute diarrhea. Further, in at least 8.5% of the total samples examined, the metagenomic approach reliably identified a different pathogen than the traditional approach. Therefore, our results provide a methodology to complement existing methods for enteric infection diagnostics with cutting-edge, culture-independent metagenomic techniques, and highlight the strengths and limitations of each approach.

## INTRODUCTION

Diarrheal disease is a leading cause of childhood mortality in low- and middle-income countries, particularly in children under the age of 5 years (1, 2), and is frequently caused by diarrheagenic *Escherichia coli* (DEC) (3–6). There are several *E. coli* pathotypes associated with diarrhea, including: Shiga toxin-producing (STEC; commonly associated with foodborne outbreaks, including O157:H7), enterotoxigenic (ETEC), enteropathogenic (EPEC), enteroaggregative (EAEC), enteroinvasive (EIEC), and diffusely adherent (DAEC) (7). Each pathotype carries distinct sets of virulence genes underlying its own mode of pathogenicity. For example, ETEC is identified by the presence of the *lt* and/or *sta* genes, which encode heat-labile and heat-stable toxins, respectively (8–10).

Effective treatment of diarrheal disease and outbreak management depends on the accurate identification of DEC strains. Cultivation from stool followed by biochemical assays and/or molecular-based methods like PCR is the conventional way of identifying some bacterial pathogens and diagnosing enteric illness (11, 12). Currently, several selective media are available for the relatively quick (12–24 h) growth of enteric pathogens. However, PCR-based amplification of virulence genes from cultured isolates is recognized as an imperfect approach to accurate diagnosis (13, 14). Further, cultivation is resource- and time-intensive, not easily scalable for hundreds or thousands of samples, and the detection of pathogen-specific virulence genes is limited to known pathotypes (14, 15). For instance, in one recent study, more than one-third of patients with traveler’s diarrhea were pathogen-negative according to culture-based diagnosis yet responded to antibiotic treatment, suggesting failure to culture the causative agent (16). Biochemical assays of cultured isolates, such as identifying lactose-fermenting strains on selective media, can support PCR-based results but only provide proof of metabolic capability, a characteristic not always linked to virulence or pathogen etiology (3). In addition, amplifying single genes from cultured *E. coli* isolates provides no information about the organisms’ genome diversity at the strain level *in-situ*, limiting its usefulness for tracking outbreaks or understanding pathogen population dynamics and epidemiology.

Whole-genome sequencing (WGS) overcomes some of the limitations of PCR and has increasingly become a useful tool in epidemiological investigations (17, 18) and in linking pathogenicity to strain-level diversity (19). However, WGS relies on cultivation and thus is limited in the ways listed above. In addition, isolation biases could affect the results and thus further diagnosis. Strains that are at low abundance (even a single cell, theoretically) and/or not the causative agent of the infection can grow on selective media, confounding interpretations. There is therefore a need to develop culture-independent methods for linking pathogen genotypes and disease outcomes, particularly for resource-limited communities where diarrheal pathogen transmission is high due to limited water, sanitation, and hygiene infrastructure.

Shotgun metagenomic sequencing provides a promising alternative to the problems associated with culture- and WGS-based approaches. By amplifying all genomic fragments within a sample, metagenomics provides an untargeted approach to assess bacterial population abundance and intra-population diversity in a host-associated sample. Recent advances in bioinformatic methods, including the recovery of a metagenome-assembled population genome (MAG) that represents the consensus genome sequence of a microbial population in the sample (20), provide both qualitative (presence/absence of pathogens) and quantitative (relative abundance) information for microbial taxa (20, 21). These approaches have been used, among other applications, to differentiate gut microbiomes of asymptomatic from symptomatic norovirus patients (22), track changes in lung microbiota of cystic fibrosis patients during treatment (23), recover genomes of *E. coli* from an STEC outbreak (24), diagnose coinfections (25), and diagnose patients with acute cholecystitis (26). As with all molecular methods, however, metagenomic-based clinical diagnosis has limitations. Most notably, low pathogen titer levels or high levels of host DNA (27, 28) can prevent adequate sequencing of the pathogen genome. Moreover, high-throughput sequencing and bioinformatic analysis can be challenging, particularly in resource-limited settings. While still far from being a standard clinical methodology, studies comparing metagenomic technologies to traditional approaches have shown that metagenomic data can be valuable for understanding disease etiology even when infections are caused by rare or difficult-to-diagnose pathogens (29, 30). Metagenomic technologies can further provide data on pathogen evolution and spread not easily attainable by traditional methods (31, 32), in part because they avoid limitations of culture-based approaches.

Current metagenomics studies have yielded genome sequences of microbial pathogens from human gut microbiomes (24, 26, 33), providing an opportunity to link pathogen genomics and disease outcomes independent of cultivation. A MAG, unlike the genome of a cultured isolate, does not usually provide strain-level resolution, particularly when multiple closely related strains co-occur and are co-assembled into a single consensus MAG, or when low abundances prevent precise assemblies. However, a MAG typically represents the most abundant genotype (or strain) within a resolved population and mapping short reads back to assembled MAG sequences can provide quantitative abundance and intra-population sequence- and gene-diversity for both pathogens and commensal gut taxa (34–36). We recently developed a bioinformatic workflow to identify the causative agent of diarrhea accompanying DEC infection by applying a combination of criteria to genome sequences of cultured isolates from a clinical sample (stool), including relative abundance of the isolate genome in the corresponding clinical metagenome based on short reads, presence/absence of virulence genes in the metagenome, and phylogenetic placement of MAGs against a reference phylogeny of the isolate genome and selected (available) genomes of pathogens and commensal relatives (37). These criteria provide a rigorous framework for accurate pathogen identification and add additional dimensions to patient data that are not obtained by culture-based approaches.

**Fig 1:**
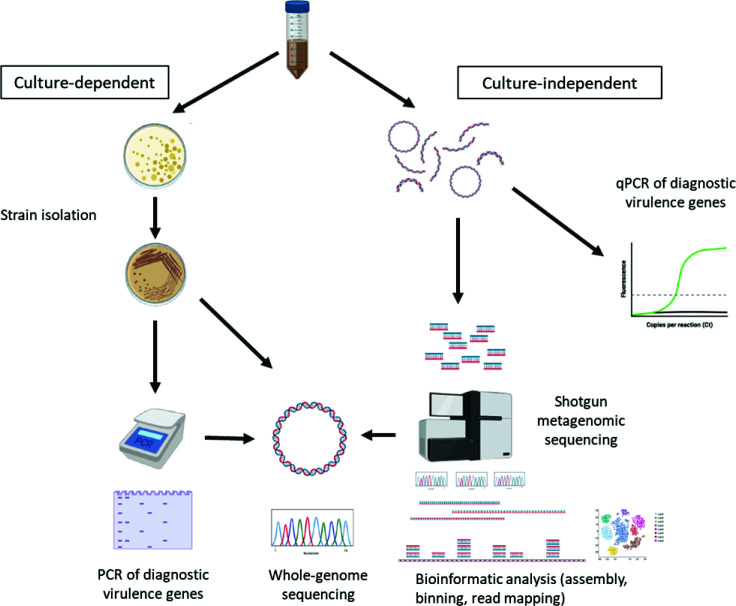
The experimental workflow guiding this study was comprised of two major tracks. The culture-dependent track included strain isolation followed by PCR of diagnostic virulence genes and whole-genome sequencing. The culture-independent track included qPCR of community DNA and shotgun metagenomic sequencing followed by metagenome assembly, genome binning, and read mapping to assembled contigs for quantitative assessment of genes and metagenome-assembled genomes (MAGs).

In this study, we compared the metagenomic sequencing workflow we described previously (37) to traditional culture-dependent approaches for DEC identification and abundance estimates (Fig. 1). We used previously collected data from a large population-based study of pathogenic *E. coli* carriage and acute diarrhea conducted in northern Ecuador (38). Our goal was to determine whether our metagenomic strategy of gene recovery via short reads and MAG binning accurately captured isolates and pathotype identities produced from the established techniques of cultivation and PCR, and whether metagenomics or cultivation provided a more accurate representation of *E. coli* pathotype populations within the gut. We recovered MAGs from a subset of samples and compared their pathotype identities to those of isolates recovered from the same samples based on genome sequencing and PCR.

## RESULTS

### Isolate whole-genome sequencing and PCR for pathotype gene identification

The human fecal samples used in this study were collected as part of the EcoZUR study, a case-control study of diarrhea and associated DEC carriage conducted in northern Ecuador from 2014 to 2015 (37, 38). Briefly, *E. coli* strains were isolated from fecal samples on selective and differential media. Five colonies, when present, were randomly chosen, pooled, and tested with conventional PCR for virulence genes (see Tables S1 and S2 for pathotype-specific primers and genes). Positive PCR led to subsequent testing of each of the five isolates individually for the corresponding virulence gene. If more than one isolate tested positive, one of the positive isolates was chosen at random for subsequent analysis and genome sequencing. From *n* > 200 samples from diarrhea cases, we selected *n* = 38 for metagenome sequencing with a PCR-identified DEC pathotype. Three of these samples were excluded due to the isolate WGS not being in our collection, resulting in a final data set of *n* = 35 isolate-metagenome pairs that were analyzed for this study.

Two sequence-based methods were used to confirm the PCR-based DEC pathotype designations. First, reference sequences of virulence genes were searched (mapped) against the assembled isolate genomes. The WGS and PCR-based pathotype identification agreed for 29/35 (83%) isolates based on this methodology (Tables S3 and S4). Six samples disagreed between WGS and PCR, these being isolates B228_2, B69_1, E184_3, E205, Q300, and B126_3. In 5/6 of these disagreements, the isolate was identified as DEC by PCR (five EPECa and one ETEC), but no virulence genes were identified in the isolate assembly. For four of these isolates, the rpoB control gene sequence was found on the assembly while the other two had no hits to any *E. coli*-specific genes in the assembly. To further compare isolate assemblies with PCR results, we BLASTed virulence gene PCR primer sequences against the assembly contigs. For 29/35, the primer pair sequences mapped to the same assembly contigs where whole virulence gene sequences were found, indicating the pathogen genes captured via PCR diagnostics were also recovered with WGS and assembly. ETEC primers and virulence genes aligned exclusively to plasmids, and DAEC primers and virulence genes were found on both chromosomes and plasmids, matching the known virulence gene locations of these pathotypes. The six remaining samples did not have any primers map to the assembly. These samples were the same six as described above, i.e., those that did not have any virulence gene matches to the assembly.

**Fig 2:**
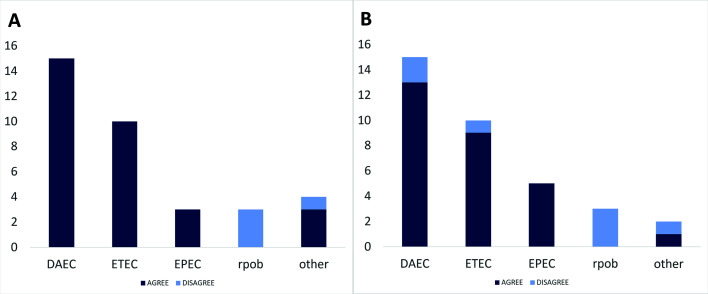
Histograms of agreements and disagreements in DEC pathotype designations based on detection of virulence genes in (**A**) PCR versus isolate WGS and (**B**) shotgun metagenomes versus isolate WGS. In both graphs, the pathotypes shown on the *x*-axis correspond to the isolate designations, and the agree/disagree condition corresponds to the PCR (**A**) or the metagenome (**B**) designations. The “other” category contains pathotypes where few samples were recovered (EAEC, EIEC, etc) or, for panel A, where the PCR and the isolate genome contain a mixed pathotype (isolate B109_1). The EPEC category contains both aEPEC and tEPEC. (**A**) WGS-based pathotype designations of isolates agreed with PCR, except for three cases where PCR outcomes were EPECa and isolate WGS contained an *E. coli rpoB* gene but no DEC genes. (**B**) There was agreement between shotgun metagenome and isolate WGS pathotype designations for 82% of samples.

To assess the possibility that the virulence genes were present in the sequenced library but were not reconstructed as part of the genome assembly, we deployed a second method to compare PCR and WGS pathotypes. For this method, unassembled short reads from isolate sequencing were searched against the reference virulence gene sequences. We recovered genes matching the PCR pathotype call in two of the disagreeing samples, bringing the matching PCR/WGS calls to 31 out of 35 (88%, Tables S3 and S4; Fig. 2A). Three of the remaining mismatches had rpoB-only hits (B228_1, E205, and R126_2), while the further sample, E184_3, had a clear match for a pathotype (EAEC) that differed from the pathotype identified via PCR (ETEC).

The recovery of virulence genes from two sequences using read mapping indicates possible issues with the assembly or sequencing process, either with the virulence genes being lost during sample processing or culture, or with the assembler failing to correctly reconstruct these regions. In the cases of disagreement, we used the genome-based identification (i.e., read mapping outcome) for the corresponding isolate for downstream analyses.

### Metagenomic recovery of *E. coli*

To determine if the cultured isolates were present in the 35 diarrheal metagenomes, we mapped metagenome reads against the corresponding isolate genome assembly. We set a high nucleotide identity threshold of 99% of reads to genome sequence to identify only the coverage of the isolate genome and avoid spurious matches to other *E. coli* genotypes in the gut. There was detectable metagenome coverage of the corresponding isolate in 31/35 (88%) based on the TAD80 metric (TAD80 ≥ 0.1*X*, Tables S3 and S4; for more details see reference 39). TAD80 refers to the truncated average sequencing depth and represents the average coverage of the genome by metagenomic reads using the middle 80% of the sequence base positions to remove outlier genomic regions in terms of coverage due to highly conserved features (e.g., rRNA genes) or high variability of sequence composition, as defined in reference 40. For these positive metagenome detections, coverage values ranged over two orders of magnitude. Of these, six metagenomes had low coverage (0.1 < TAD80 < 1.0) of the isolate genome in the corresponding metagenome (Table S3), indicating that the isolate represented a minor member of the microbiome.

**Fig 3:**
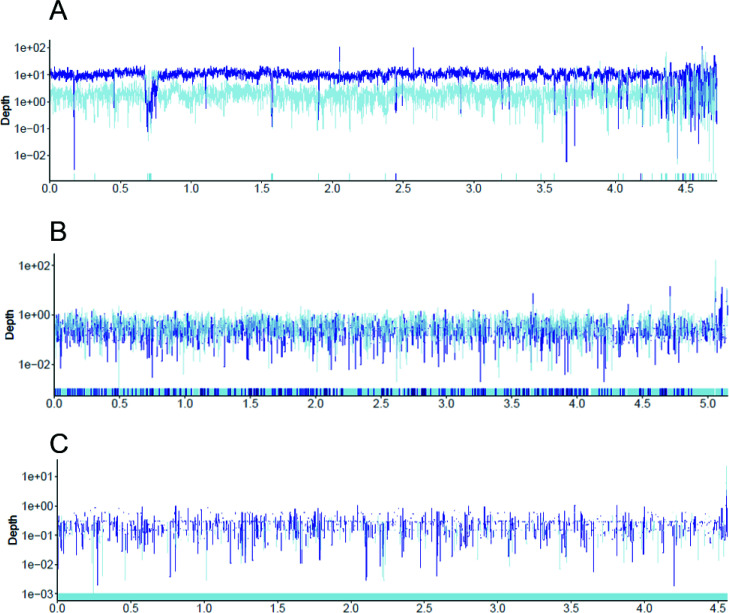
Recruitment plots of isolate genome coverage in the corresponding metagenome for (**A**) isolate B295_2 and metagenome MG_16, (**B**) isolate B228_2 and metagenome MG_14, and (**C**) isolate Q300 and metagenome MG_36. Dark blue lines represent coverage depth (*y*-axis) by reads that map at 99% nucleotide identity or above across the genome (*x*-axis). Light blue lines indicate reads mapping at identities lower than 99% identity, indicating the presence of closely related *E. coli* populations. For B295_2/MG_16 (**A**), the isolate is well-represented in the metagenome, as indicated by many dark blue bars mapping with high identity. For B228_2/MG_14 (**B**), the isolate maps at the limit of detection, with co-presence of another abundant *E. coli* population in the metagenome. For Q300/MG_36 (**C**), sparse coverage of the isolate is observed indicating this isolate sequence was at the limit of detection and borderline undetected in the metagenome.

An additional four samples that had TAD80 scores at or lower than 0.1 were inspected manually using read recruitment plots. This was done to establish the presence or absence of the isolate at the sequencing effort applied (Fig. 3). TAD80 is a conservative metric that produces no false-positive results, but may produce false negatives, which we sought to confirm via manual recruitment plot inspection. These plots give a visual representation of depth and breadth of coverage of reads in a genome. Metagenomes MG_10, MG_14, and MG_5 had TAD80 scores ranging from 0.01 to 0.07, and MG_36 had a TAD80 score of 0. One of these, MG_14, had an isolate genome-based pathotype identification that disagreed with the PCR result as described previously. Based on read recruitment plots, we determined that MG_10 had strong detection of the isolate at >99% nucleotide identity. MG_14 and MG_5 had weak, if any, isolate detection at >99% nucleotide identity. In addition, the latter two samples appeared to have other *E. coli* populations at lower identity thresholds (Fig. 3, light blue lines), indicating substantial intrapopulation diversity. MG_36 had sparse coverage of the isolate genome at all identity levels. We therefore determined that metagenome sequencing of MG_10, MG_14, and MG_5 detected the cultured isolate, at the limit of detection of our sequencing effort while MG_36 was at or below the limit of detection, and thus this sample was designated as “undetected” by metagenome sequencing. This resulted in an overall 34/35 (97%) detection of the cultured isolates by their corresponding metagenomes across several orders of magnitude of relative abundance.

### Read-based pathogenic *E. coli* gene detection in metagenomes

Given that metagenome sequencing reliably detected an *E. coli* population with high identity to the isolated strain in almost all samples, we next examined whether the virulence genes identified in the corresponding isolate by PCR/WGS were also present in the metagenome by searching short metagenomic reads against DEC virulence genes. Because metagenome data are compositional, we recorded any virulence genes detected based on 0.1× read coverage threshold, with any borderline or lower coverage cases further examined visually using recruitment plots (Fig. S1). This was done to ensure that virulence genes within the metagenome would not be missed due to low relative abundances (Table S3, see Materials and Methods for in-depth description).

We detected a pathogenic DEC gene in 97% (34/35) of the metagenomes. A single metagenome had no DEC virulence genes, even though an *E. coli* population was detectable for these samples (Tables S3 and S4, Fig. 2B). Of the positive detection group, 28 (82%) metagenomes had genes that corresponded to the DEC isolate pathotype from the same sample. Of these, 16 (57%) also had virulence genes associated with another pathotype. For the seven metagenomes that we did not detect virulence genes corresponding to the isolate, there were three types of disagreements between the pathotype identity of the isolates and metagenomes. These were either (i) the metagenome contained virulence genes when such genes were not detected in the isolate (only *rpoB* detected, three cases), (ii) the metagenome had no virulence genes detected, while the isolate was designated as DEC (one case), or (iii) mismatch of the DEC pathotypes detected in the isolate vsversus the metagenome analysis (three cases, Fig. 2B). We found that in the first case, the metagenomes detected EPECa, DAEC, and EIEC virulence genes. We cross-checked this with the TAD80 scores of the metagenomic reads aligned to the isolate assemblies at >99% nucleotide identity, since these scores indicate how well the isolate strain is covered by the metagenome. We found that for these three samples, TAD80 scores of metagenomic reads to the isolate were 0.07, 0.62, and 1.13, respectively, at >99% nucleotide identity (strain level), versus TAD80 scores of 0.25, 0.95, and 1.56 at >95% nucleotide identity (species level). These TAD80 differences at strain versus species-level ANI thresholds indicate that these specific isolates were likely not the dominant members of the overall *E. coli* population. For the samples where metagenomic reads aligned to different DEC virulence genes than their corresponding isolate genomes (disagreement type 3 above), we found a similar trend of low coverage of the isolate in the metagenome, with TAD80 scores of these ranging from 0.1 to 0.21, though one sample had high isolate coverage of 28.25. The low TAD80 scores of metagenomic reads compared to isolates in these cases again likely indicate that the recovered isolates are minor members of the microbiome. We also observed that 4/7 disagreements between the metagenome and isolate pathotypes were from samples that also had conflicts between the PCR and isolate WGS DEC pathotype designations, where the isolate assembly either did not include a diagnostic DEC virulence gene profile or where the pathotypes did not agree.

### Co-occurrence of multiple *E. coli* populations in metagenomes

As a final step of investigating DEC identities and *E. coli* populations within the metagenome, we examined whether there was more than one closely related *E. coli* genotype (or sub-population) co-occurring in the microbiome. Low abundances of commensal *E. coli* strains are common in healthy gut communities. These commensal *E. coli* often proliferate during pathogen infection due to changes in the gut that make conditions more hospitable for *E. coli* (25), and they can be challenging to distinguish from pathogenic strains (41). We were particularly interested to know if *E. coli* populations from samples with low TAD80 scores for isolate coverage in the metagenome were better represented by a commensal genome, or if there was evidence for a pathogen other than the isolate causing diarrhea symptoms. To this end, we used a competitive read recruitment approach, competitively blasting metagenome short reads to both their matching isolate assembly and a commensal *E. coli* genome representative, strain HS (NC_009800.1), and calculating TAD80 scores at a threshold of >99% nucleotide to distinguish between closely related sequences. We found three metagenome samples where TAD80 coverage was higher for the commensal than the matching isolate assembly, with TAD80 scores of 8.39 versus 3.4 (MG_21), 2.24 versus 0.26 (MG_22), and 1.93 versus 0.23 (MG_23). We examined these three samples with recruitment plots and found that while both commensal and isolate genomes had detectable coverage by the metagenome, there appeared to be higher coverage of certain regions of the commensal genome compared to that of the isolate, particularly in MG_21 and MG_22. In MG_31 and MG_32, both the commensal and the isolate genomes were equally well-covered by metagenomic reads. For MG_31, relative abundances of the commensal and isolate genomes were very similar, constituting 22.16% and 21.05% of the metagenome, respectively.

### Metagenome-assembled genome recovery

We binned MAGs from the 35 metagenomes to compare the dominant *E. coli* population from the metagenomes to the corresponding isolate pathotype genomes. MAG binning resulted in 13 high-quality (i.e., assembly completeness − 5× contamination score, with scores <50 discarded) *E. coli* MAGs from 13 samples. All MAG-associated samples had high metagenome coverage of the corresponding DEC isolates (average TAD80 scores of 33.77, ranging from 3.26 to 106.03) and 12/13 MAG-associated samples had agreement between metagenome and isolate-based analyses of diagnostic DEC virulence genes (i.e., matching pathotypes), demonstrating that these genes had been recovered sufficiently at the read level within the unassembled metagenome. Despite this, only 38% of *E. coli* MAGs (5/13) contained the same virulence genes as the isolate genome from the same sample. Both the MAGs and the corresponding isolate genomes for these five samples contained virulence gene profiles indicative of DAEC. For the remaining eight samples, no virulence genes were found in the MAG assemblies (Table S6). However, seven of these eight samples had (unassembled) metagenomic reads that matched multiple *E. coli* virulence genes, indicating possible multiple *E. coli* populations present in the samples that likely interfered with MAG assembly and/or binning.

### MAG versus isolate clonal identities

**Fig 4:**
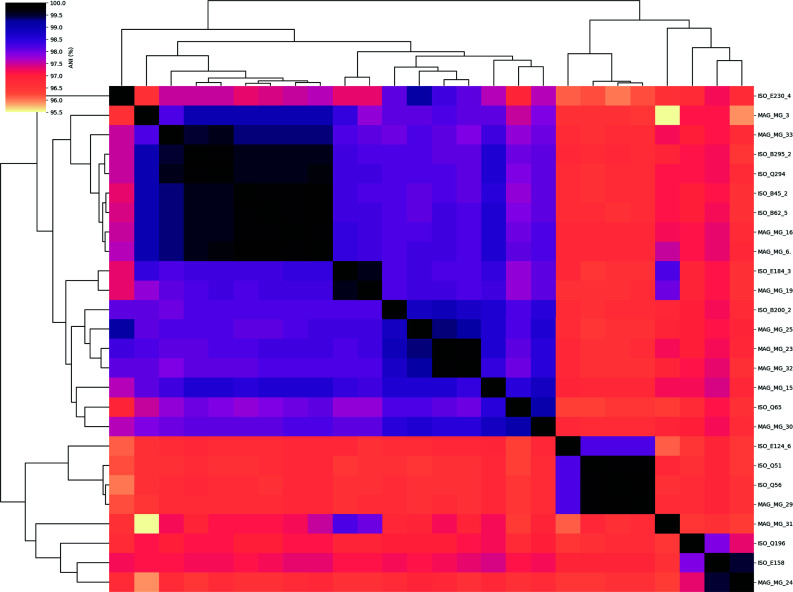
Relationships between assemblies of MAG-isolate pairs. ANI matrix shows all-against-all ANI distances for the entire assemblies of the same MAG-isolate pairs. Black boxes indicate 100% identity between samples.

A major focus of our study was to determine if the recovered MAGs represented the isolate obtained in culture from the same sample, both in terms of clonal identity (i.e., whether the MAGs captured the exact genotype represented by the isolate) and pathogen identity (i.e., pathogenic gene content). We measured clonal identity by calculating ANI between the 13 MAG-isolate pairs. ANI values ranged between 96.8% and 99.9% (Fig. 4), indicating that some pairs were not the same genotype (e.g., when showing <99.5% ANI).

**Fig 5:**
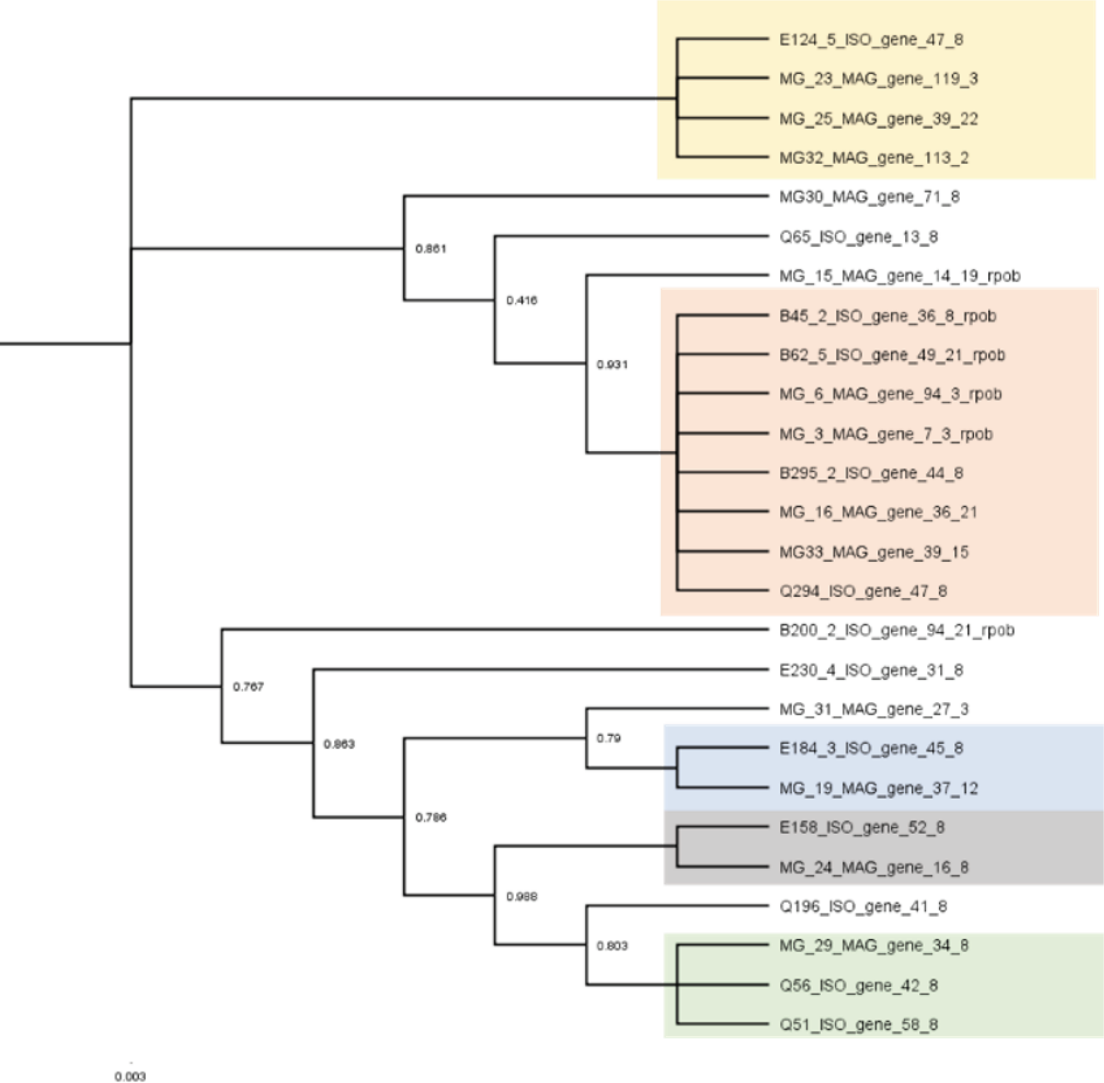
Phylogenetic tree of *rpoB* genes extracted using MiGA from MAG-isolate pairs from the 13 samples where high-quality *E. coli* MAGs were assembled. Color-filled boxes behind terminal branches indicate clonal groups where *rpoB* genes are 100% identical.

To further compare *E. coli* MAGs and isolates, we extracted *rpoB* sequences for phylogenetic analysis. Of the 13 MAG-isolate pairs, 8 (61%) had clonal (identical) *rpoB* sequences, with the rest having varying degrees of sequence identity (Fig. 5). Phylogenetic analysis revealed five distinct *rpoB* clonal groups (i.e., identical *rpoB* sequences within a group), with the largest group being composed of four different MAG-isolate pairs. The remaining groups consisted of single pairs (two groups) or a single pair plus additional (singleton) MAGs or isolates from unrelated pairs (two groups). From these results, we observed that while MAGs and their corresponding isolates were closely related strains of *E. coli*, they were not clonal in many cases based on the *rpoB* sequence analysis (about half of the total), and that there were several *rpoB* clonal groups residing within individual patients, which warranted closer investigation.

### Pathotype of MAGs versus isolates

To this end, we compared pathogenic identities between MAG-isolate pairs. Of the 13 samples that yielded a high-quality *E. coli* MAG, the only virulence genes that were detected in MAG contigs were associated with DAEC, which occurred in 5/13 MAGs. These five MAGs matched the DAEC isolate pathotype designation from the same sample, with isolate coverages in the metagenomes averaging 47.3 (TAD80). Interestingly, only two of the five MAG-isolate pairs with matching pathotypes had clonal *rpoB* gene sequences (Fig. 5 and 6; Table S6) and ANI between MAGs and isolates of these five matching pathotype pairs ranged between 96.9% and 99.9%. In the remaining eight MAG-isolate pairs, the *E. coli* MAG did not match the diagnostic virulence gene profile and DEC pathotype designation of the isolate. Based on the genome sequence, the corresponding isolates for these eight pairs were designated as DAEC (two pairs), ETEC (five pairs), and EAEC (one pair), with TAD80 scores of the isolates in the metagenomes averaging 25.3. We noted that several metagenome short reads reliably mapped to the virulence genes identified by the isolate WGS, with an average TAD of 18.63, indicating that the virulence genes had been sequenced but were not assembled and/or binned as part of the MAG sequence. qPCR gene copy numbers for virulence genes in DNA extracts from whole stool were also plotted against the TAD80s of MAGs from the same samples and showed that the DAEC-associated *afa* copy numbers increased as MAG TAD80 increased, indicating positive correlation with copy number to MAG abundance. There was no relationship between ETEC-diagnostic *lt/sta* genes by qPCR and MAG TAD80 values (Fig. S2A through C).

Because of the high clonal identity between MAG-isolate pairs and the reliable coverage of virulence genes within the metagenomes of these eight samples, we wanted to further corroborate that the metagenomic reads that mapped to virulence genes were simply not assembled as part of the MAG due to limitations of the assembly and/or binning steps. We were especially interested in whether the corresponding reads were present on plasmids or other mobile elements since these can be challenging to bin with chromosomal contigs (42). To investigate this, we extracted the individual contigs from the isolate assemblies where DEC diagnostic virulence genes aligned and ran them through NCBI blast. ETEC virulence genes in all isolate samples were located on contigs with high sequence identity to known *E. coli* plasmids (Table S9). DAEC-associated virulence genes were located either on chromosomal genomic islands or plasmids and we found that the two DAEC MAG-isolate pairs where the MAG did not recover DAEC had DAEC genes on chromosomal contigs.

### Quantification of *E. coli* pathotypes in metagenomes based on read placement

To further corroborate these results, we calculated the proportional representation of each MAG in each metagenome relative to the total *E. coli* population using competitive read recruitment of metagenome short reads to MAG and isolate *rpoB* sequences. Competitive read recruitment maps sample reads to one or more genomes, and the best genome match for each read is reported via custom scripts. Eight of the 13 *E. coli* MAG/isolate pairs had clonal *rpoB* sequences (Fig. 5 and 6) and nucleotide sequence identity of these pairs were all >99%, indicating the same strain types were likely captured. For the other five pairs with different *rpoB* alleles, competitive read recruitment indicated alleles of the five MAGs were more abundant than those of the corresponding isolates (including in two metagenomes with DAEC isolates but no corresponding DAEC MAGs, Table S6). These results are further confirmation that the most abundant *E. coli* strains are not always captured in culture and are more thoroughly represented via metagenome data.

## DISCUSSION

The technological advancements and decrease in the cost of shotgun metagenomic sequencing have opened new opportunities for rapid and informative assessment of diseased gut microbiomes. For diarrheal disease, which can be caused by several different pathogens, accurate diagnosis is imperative for guiding treatment and understanding outbreak dynamics. Isolation of *E. coli* pathogens with PCR ID can be accurate and definitive but has several limitations that preclude a full understanding of disease etiology with the potential to lead to incorrect diagnoses. In this study, we evaluated the ability of both culture-dependent PCR and culture-independent metagenomic sequencing methods to identify pathotypes of DEC strains colonizing the gut.

Metagenomes from 34/35 (97%) of samples had detectable read coverage of their corresponding isolate genomes, indicating that metagenomic sequencing reliably detected the isolates obtained from culture. For 4 of these 34 samples, the isolate was at relatively low relative abundances, preventing its positive detection by the automated TAD80 score approach (score > 0), and requiring visual inspection of read recruitment plots (Fig. 2B). Therefore, manual inspection may be required for detection of targets at or just below the limit of detection of the sequencing effort applied. Approaches to estimate the latter based on a similar methodology to that employed herein have also been recently reviewed (43).

Notably, read recruitment plots also showed that while most isolates matched metagenomic reads at high identity (>99% nucleotide identity), there were often mixed populations of closely related *E. coli* within the metagenomes (Fig. 3A through C), and 16/35 (46%) of metagenomes had additional *E. coli* virulence genes that did not match the pathotype of the isolate. The variable isolate abundances in the metagenomes, mixed populations of *E. coli*, and evidence of multiple DEC pathotypes in the metagenomes support our conclusion that cultured isolates are not necessarily representative of the most abundant *E. coli* population *in situ* for most of these 16 samples. However, it was not always possible to conclude that the alternative pathotype detected by metagenome read mapping to diagnostic genes was indeed the causative agent (as opposed to the pathotype detected by culturing). This was because the metagenomic results could be attributed to technical limitations in DNA extraction and downstream processing between the WG and metagenome sequencing. Specifically, we used 0.1–0.5 g of stool to extract DNA for metagenomes, and it is possible that low-abundance and/or spatially heterogenous populations of DEC were not captured using this small sample mass. The timing of sample collection may play a role in differences, we detected between metagenome and WGS as well. Samples were collected from patients reporting acute diarrhea in clinical settings, and stool collection may have occurred when pathogens were being cleared and were at lower abundance. DNA extraction from larger sample volumes or longitudinal stool collection that captures the peak of infection might address these concerns. Nonetheless, the large number of samples with multiple *E. coli* populations detected (46% of the total) indicated that at least some of these cases were not simply attributed to technical limitations but truly represented the population in the sample. Moreover, in at least three of these cases (8.5% of the total 35 samples), we accumulated strong evidence that the isolate pathotype (DAEC) was most likely not the causative agent, and a different, more virulent and abundant pathotype was detected by the metagenome instead. Accordingly, we suggest the use of shotgun metagenomic sequencing together with traditional isolation-based diagnostics for a more complete picture of DEC infection and difficult-to-diagnose cases in the future.

A major objective of our study was to determine if *E. coli* MAGs could be used as accurate representations of cultured isolates and their pathotype identities. ANI distances between MAG-isolate pairs, particularly in terms of nucleotide identity between extracted *rpoB* sequences, demonstrated high intrapopulation diversity of *E. coli* populations in some samples, with clonal groups frequently found across samples rather than shared by a MAG-isolate pair within a single sample. Pathotype designations differed between the two methods for 8/13 (62%) MAG-isolate pairs (Table S6), but the cause and the frequency of the discrepancy differed between samples associated with DAEC versus ETEC isolates.

For DAEC, MAG assembly and binning largely agreed on the isolate genome pathotype mostly because the diagnostic genes are usually found as single-copy on the chromosome, which is typically not challenging for assembly and binning. DAEC has been epidemiologically associated with diarrheal disease in children but is rarely identified as the causative agent of diarrhea in adults (44, 45). In fact, adults often remain asymptomatic for acute diarrhea following challenges with DAEC strains (46, 47). Thus, the isolation of DAEC pathotypes from adults with DEC infection may be a false positive signal of the causative agent, according to several studies. The latter is consistent with our observation that 12/20 metagenomes with reads that recovered DAEC virulence genes also recovered virulence genes from other pathotypes of DEC (Table S4). Therefore, MAGs appear to provide reliable identification of DAEC infections, including in cases, where DAEC is at low abundance and might not be the primary pathogen.

In contrast, MAG binning was less effective at representing ETEC populations in the gut. ETEC genes were recovered at the read level but were consistently absent from corresponding MAG assemblies, presumably due to their location on plasmids (Tables S5 and S9) (8–10). Plasmids are known to escape metagenomic binning due to their variable copy numbers and sequence composition, which can be different from chromosomal DNA and thus, be challenging for binning algorithms (42, 48). Therefore, in cases where virulence genes are associated with plasmids such as those that define the ETEC pathotype, mapping short reads to pathogenic gene sequences, rather than assembly and binning of isolate genomes, could be used for pathogen identification. Metagenomic binning of ETEC populations can still be highly informative, however, even when virulence genes are absent from the resulting MAG. For example, phylogenetic placement of the MAG among other available ETEC genomes could indicate the presence of an ETEC MAG even if virulence genes cannot be found as part of the MAG sequence. Additional manual examination of contigs and their coverage patterns as performed here and/or long-read sequencing for more reliable assembly and genome binning and/or manual curation may also prove useful for identifying plasmid-based virulence genes. Cultivation of ETEC strains can also be effective, as isolates generally retain the plasmid containing *eltA/eltB/sta* virulence genes in culture based on our data. Cultivation and metagenomics approaches seem to be highly complementary for ETEC infections.

For our 35 samples, we found 88% agreement in the diagnostic virulence gene-based DEC pathotype designation between PCR and WGS of cultured isolates. Cultured isolates from four samples that yielded EPECa virulence genes via PCR of extracted DNA contained neither PCR primer nor pathogenic gene sequences. This discrepancy suggests that the genes were present in the isolated strains but were subsequently lost from the genomes during the culturing process or error during PCR analysis. The loss of these genes due to location on plasmids is most probable, especially because genomes of matching isolate genotypes deposited in NCBI showed plasmid rather than chromosomal locations for the same genes (Table S5). That is, differences in the template DNA used for the two methods may account for the loss of these genes, since PCRs were performed on plate colonies, while DNA for WGS was extracted with a Wizard Genomic DNA Purification Kit from liquid cultures grown from separate (subsequent) colonies (Promega, Madison, WI). Nonetheless, incomplete genome sequencing cannot be excluded as a possibility for these disagreements although this scenario appears to be comparatively less likely because the genomes were sequenced at high coverage, and we employed a read-based approach to deal with assembly artifacts and limitations. These discrepancies encompass common issues with culture-based approaches, including loss of plasmid-encoded virulence genes during culturing, use of different processes and kits for PCR extraction versus WGS, or effects of freezing/thawing multiple times for repeated PCRs and assays.

### Conclusions and future directions

Metagenomic approaches provide crucial context for DEC and other enteric diseases beyond the information provided by cultured isolates. Insights into the relative abundances of target strains and identification of co-occurring strains obtained by metagenomics could inform researchers and clinicians of the makeup of pathogenic *E. coli* populations in patients suffering from diarrhea or other intestinal disorders. In this study, we demonstrated the use of a combination of read-based DEC diagnostic virulence gene recovery, manual recruitment plot examination, and MAG analysis was an advantageous approach to obtaining these metrics, and complemented information obtained by PCR. Notably, this approach can be easily extended to other pathogenic lineages of enteric bacteria including *Salmonella* and *Campylobacter*. The pathotypic identity of the present *E. coli* played an important role in the effectiveness of our metagenomic workflow to detect DEC, however; and we found that different pathotypes warranted different analytical approaches. Specifically, DAEC infections were readily identified via read mapping to virulence gene sequences and frequently MAGs, while ETEC infections were readily identified by read mapping to virulence genes but not MAGs. The difficulty of identifying ETEC infections by MAG recovery alone highlights some of the shortcomings of bioinformatic approaches, namely the computational challenges associated with identifying and binning plasmid-encoded virulence gene sequences. MAGs also theoretically represent the most abundant members of the community (because higher read abundances provide better bins in general); in practice, there are genome features (e.g., plasmids and high intra-population diversity) that can prevent effective binning, even when the population is highly abundant (41, 42). Recent technological advances, however, such as rapid long-read sequencing like Nanopore technology and strain-level binning (49), suggest that these approaches will become an important component of clinical diagnoses (e.g., references 50, 51). There is therefore a need for versatile metagenomic pipelines that incorporate both newer sequencing technologies and traditional cultivation-based methods.

In summary, our work has demonstrated that while there are certain limitations to metagenome sequencing for DEC identification, they may be overcome with a combination of bioinformatic analytical approaches, which are continually improving. This work has also contributed toward the standardization of bioinformatic pipelines by assessing different metrics (e.g., TAD80) and highlighting areas for improvement in genome binning and coverage metrics. Deployment of this analytical pipeline using workflow engines such as NextFlow (52, 53) can further speed up and streamline analysis in resource-limited settings. Overall, we conclude that a combination of both gene recovery at the read level and MAG analysis are effective at identifying DEC in human microbiome samples, providing a valuable next step in the path to optimizing DEC diagnostics as well as complementing existing methods.

## MATERIALS AND METHODS

### Study design and sample selection

Subjects (*n* = 907) were enrolled in EcoZUR, a case-control study of diarrhea in Northern Ecuador as described in reference 38. A total of 771 participants submitted stool samples which were subjected to both cultivation-dependent and cultivation-independent analyses (Fig. 1). These were assayed by (i) PCR for virulence genes and (ii) whole-genome sequencing followed by bioinformatic analysis of diagnostic DEC virulence genes (*n* = 213 positive for DEC by PCR). Further, community DNA from the stool samples was extracted and used for (i) qPCR of diagnostic virulence genes and (ii) short-read shotgun metagenomic sequencing followed by bioinformatic analysis. The subset of samples (*n* = 35) included in this study were selected based on the following criteria: (i) the sample was from a participant who presented with diarrhea (a diarrhea case sample, defined as three or more loose stools in 24 h), (ii) there was an *E. coli* strain isolated from the sample that was PCR-positive for a pathogenic DEC gene profile, and (iii) we had a shotgun metagenome available for the sample from prior analyses (4, 37, 54). Details of each workflow are provided below, and further details about study design, sample selection, and processing, and for culturing, isolate sequencing, and shotgun metagenome sequencing can be found in references 4, 37, 38, 54, 55.

### *E. coli* strain isolation and PCR of virulence genes

*E. coli* strains were isolated from fresh stool samples using MacConkey’s agar media followed by Chromocult agar media to test for β-glucuronidase activity and confirmed with biochemical tests as described in reference 38. Five *E. coli* colonies per sample were chosen for pathotype identification using a pooled conventional PCR method. Template DNA was obtained by pooling the five colonies, resuspending them in 300 µL sterile distilled water, and boiling for 10 min to release DNA. The resulting supernatant was used to identify *E. coli* pathotypes by singleplex conventional PCR assays. If a pooled sample tested positive for any virulence factor, then each of the five isolates was retested individually to identify the gene(s) carried by each isolate. Any isolates with virulence genes were kept for downstream processing, resulting in multiple isolates analyzed from single fecal samples in some cases. *E. coli* pathotype identification by conventional PCR was determined using the following virulence gene criteria: *bfp* and *eaeA* for EPEC (atypical EPEC is *bfp*+/*eaeA*−, typical EPEC is *bfp+/eaeA*+), *It* and/or *sta* for ETEC, *ipaH* for EIEC and Shigellae*, aggR* for EAEC, and *afaBC* for DAEC (37) (Tables S1 and S2).

### DNA extraction of isolates and stool samples

*E. coli* isolate DNA was extracted from fresh cultures using the Wizard Genomic DNA Purification Kit (Promega, Madison, WI) according to the manufacturer’s protocol. DNA for stool metagenomes and qPCR was extracted from 0.2 mL homogenized stool using the MoBio (now Qiagen) Powersoil DNA isolation kit according to the manufacturer’s protocol. A NanoDrop spectrophotometer (Thermo Scientific) and a Qubit 2.0 dsDNA high-sensitivity assay (Invitrogen, Carlsbad, CA) were used to estimate the purity and concentration of both isolate and whole stool DNA extracts.

### Whole genome and metagenome sequencing

Isolate genomes and fecal metagenomes (*n* = 35, for samples that yielded pathogenic *E. coli* isolates) were sequenced as previously described (37). Briefly, both libraries were prepared using an Illumina Nextera XT DNA library preparation kit and quantified using the Qubit dsDNA HS assay (ThermoFisher) and run on a high-sensitivity DNA chip using a Bioanalyzer 2100 instrument (Agilent) to determine library insert sizes. An equimolar mixture of the isolate libraries was sequenced on an Illumina MiSeq instrument using a v2 500-cycle reagent kit (2 × 250 bp paired-end run; Illumina, Inc.) at a final loading concentration of 10 pM. Metagenomic libraries were sequenced on an Illumina HiSeq 2500 instrument using a 2 × 150 bp paired-end kit at 300 cycles.

### Isolate genome assembly and metagenome population genome binning (MAGs)

Human reads were removed from raw metagenomic sequences using BMTagger (56). Human read-decontaminated metagenome (59) and isolate genome (279) sequence reads were trimmed and assembled using the MiGA (Microbial Genomes Atlas) pipeline for metagenome sequences (57). MaxBin2 (58) was used to *de novo* bin assembled metagenome contigs >2 kbp in length into MAGs. MAG quality was evaluated using the CheckM v1.0.3 (59) *E. coli* taxonomy-specific workflow. MAG quality scores were calculated as estimated completeness minus five-times estimated contamination, and MAGs with scores <50 were discarded. The taxonomy of high-quality *E. coli* MAGs and isolate genomes was confirmed against a reference database using MiGA’s average nucleotide identity (ANI) and average amino acid identity (AAI) based methods. MyTaxa scan barplots (60) and MiGA estimates of genome contamination and completeness based on lineage-specific marker genes were used to further confirm taxonomy and assess the quality of the recovered *E. coli* MAGs.

### Average nucleotide identity for isolate/MAG pairs

ANI values were calculated for each *E. coli* isolate genome and the corresponding *E. coli* MAG (binned from the same sample from which the isolate was cultured) using ANIclustermap (61), which is based on FastANI (62). ANI matrices and heat maps were generated with ANIclustermap.

### Bioinformatic-based isolate genome and MAG pathotype designations

To classify pathotypes of isolate genomes and MAGs, pathotype virulence genes (Table S2) were queried against the assembled contigs using BLASTn with the parameters “— perc_identity 40” and “—task blastn.” These parameters change the default pre-alignment filter from megablast (large word size) to blastn (word size of 7), thus retaining any matches with a relatively lower percent identity.

Trimmed reads from isolated whole-genome sequences were queried against all virulence genes using Magic Blast (63). Virulence genes were classified as present when sequencing depth was above 0.1 for recovery with short reads. If at least half of the virulence genes of a single type were identified but coverages for these sequences were below 0.1, or if less than half were below 0.1 with some reads at 0.1 and above (mixed), the sample was marked as having the pathogen at low coverage. Single genes with coverages below 0.1 for any pathotype were not considered positive at the limit of detection, and were manually inspected using read recruitment plots generated with RecPlot4 (available at https://github.com/KGerhardt/Recplot_4).

### Metagenome relative abundance of isolate genomes and MAGs

Relative abundances of isolate genome sequences and MAGs in metagenomes were calculated by aligning trimmed reads to genome contigs with Magic Blast. Alignment results were used to calculate the 80% truncated average sequencing depth (TAD80) with the Enveomics “seqdepth” script (64). TAD80 values were normalized by the genome equivalent value of the corresponding metagenome (64) for a final relative abundance value as previously described (40).

Trimmed metagenome reads were mapped to isolate whole-genome sequences using Magic Blast and the results were combined with MAG contigs to build recruitment plots showing coverage across the genome. We assessed the coverage of individual genes using custom Python scripts (available at https://github.com/rotheconrad/00_in-situ_GeneCoverage) and calculated values using reads with at least 99% nucleotide identity to the reference MAG. This identity threshold represents high stringency to capture only sequences with extremely high, strain-level identity to the reference sequence, contrasting with a threshold of 95% nucleotide identity to capture species-level read matches as defined in reference 65.

### Metagenome relative abundances of DAEC and ETEC marker genes

Relative abundance of DEC diagnostic virulence gene sequences in metagenomes was calculated by aligning short reads to gene sequences with Magic Blast as described above. The reference gene sequences and diagnostic criteria for each pathotype (DAEC and ETEC) are provided in Table S2. Results were filtered by read length (minimum length 70 bp) and alignment length/read length (minimum 0.7) using the Python script 01 c_MagicBlast_ShortRead_Filter.py. TAD80 values were calculated using the Python script 03 a_MagicBlast_CoverageMagic.py without excluding coverage outliers (-*d* parameter 100) to include all reads with identity above the threshold. Scripts can be found at https://github.com/rotheconrad/00_in-situ_GeneCoverage. For EAEC, the average TAD80 of the two virulence genes (*lt*A and *lt*B) was used as the coverage value. For DAEC, because 32 genes were queried and often not all genes were present, the average TAD of all values above 0.1 was used.

Unbinned metagenome contigs whose source samples yielded ETEC isolates but whose corresponding *E. coli* MAGs contained no DEC diagnostic virulence genes were searched for the ETEC virulence genes *lt*A and *lt*B using Magic Blast. Open reading frames on each contig were identified using Prodigal and annotated using Prokka (66) and BLAST against the NCBI nr database. Contigs were classified as plasmids if they (i) contained transposases or mobile genetic elements, and (ii) if the top BLAST matches against non-redundant (NR) database of NCBI included known *E. coli* plasmids.

### Determining *E. coli* genotype and abundance levels in metagenomes

*rpoB* genes of MAGs and isolate genomes were extracted using the MiGA webserver (57) and concatenated for competitive recruitment of trimmed metagenome reads using Magic Blast (63). A gene phylogeny was generated by aligning the *rpoB* sequences with Mafft (67), running the alignment in Fasttree (68) using a maximum-likelihood model, and visualizing the results with Figtree (available at https://github.com/rambaut/figtree).

Coverages of non-clonal *rpoB* genes from each metagenome-isolate pair were assessed by competitively aligning unassembled reads of metagenomes and their corresponding isolates against the extracted *rpoB* full sequences from isolate and MAG genomes using Magic Blast. The output was filtered by a minimum read length of 70 nt and 100% identity.

### qPCR of DAEC and ETEC marker genes

For a limited number of samples for which DNA remained following metagenomic library preparation and sequencing, quantitative PCR (qPCR) was used to quantify total bacteria, total *E. coli*, and DAEC and ETEC gene markers for a subset of samples for which sufficient DNA template from whole stool samples remained after metagenomic sequencing library preparation. The assay gene targets were *daa*C (DAEC) and *st* and *lta* genes (ETEC) (Table S2) (69–71). For all assays, samples were run in duplicate on a Bio-Rad CFX96 real-time PCR system. About 20 µL final volume reaction mixtures contained 10 µL of 2× SYBR Select Master Mix (Applied Biosystems, Austin, TX), 0.25 µM each forward and reverse primer, and 4 µL of DNA template. Cycling conditions for all assays were as follows: 50°C for 2 min, 95°C for 2 min, and 40 cycles of 95°C for 15 s, 6–61.5°C for 15 s, and 72°C for 1 min. Primers and annealing temperatures for assays used are summarized in Table S1.

Gene abundance was quantified by interpolation to a standard curve as the mean concentration of duplicate reactions and reported as gene copies per ng DNA template.

The standard curve was generated using gBlock gene fragments (Integrated DNA Technologies, Coralville, IA) that contained the target sequence at 10-fold dilutions ranging from 10^6^ to 10^1^ gene copies per reaction. Negative controls (no template added) were included on each qPCR plate. An inhibition control assay was used to test for qPCR inhibition, and 2.5 × 10^4^ copies of an artificially designed inhibition control gene target (72) were spiked into all samples. *C*_*t*_ values of the inhibition control recovered from all samples were compared to those recovered from spiked nuclease-free water as a benchmark. No sample inhibition (defined as a *C*_*t*_ value difference >2) was detected in the samples.

Standard curves were analyzed according to published Minimum Information for Publication of Quantitative Real-Time PCR Experiments (MIQE) guidelines (73, 74). Detection and quantification methods are reported as described in reference 75. Limits of detection were defined as the lowest amount of template that could be reliably detected above the negative control for each assay run. The limit of quantification was defined using the standard curve as the gene target concentration where the standard deviation for all replicates was less than or equal to 2 *C*_*t*_ values. Results were quantified if the duplicate reactions were both amplified, fell within two standard deviations of each other, and were above the level of the lowest standard. If zero or one well was amplified, the result was deemed ND and designated a value of half the limit of detection. If both duplicates were positive, but amplification occurred after the lowest dilution, the result was considered detected but not quantifiable (DNQ) and assigned the value of the limit of detection. Average assay efficiency was between 95% and 105%, with the exception of the total bacteria assay which had an efficiency of 87.5%.

## SUPPLEMENTAL MATERIAL

Supplemental material mbio.03422-23-s0001.pdf Supplemental tables and figures.

ASM does not own the copyrights to Supplemental Material that may be linked to, or accessed through, an article. The authors have granted ASM a non-exclusive, world-wide license to publish the Supplemental Material files. Please contact the corresponding author directly for reuse.
