# Supplementary material for: Comparison of metagenomic and traditional methods for diagnosis of E. coli enteric infections
Source: mBio. 2024 Mar 15;15(4):e03422-23. doi: 10.1128/mbio.03422-23 (PMC11005377; doi:10.1128/mbio.03422-23)
Supplement: Supplemental material — Supplemental tables and figures. [file mbio.03422-23-s0001.pdf]

Supplementary Figures and Tables

Supplementary Table 1: Primers used to detect diarrheagenic *E. coli* virulence genes in *E. coli* isolates by conventional PCR [71-74].

Gene	**Primer sequence 5 – 3’**	**Size (bp)**	**Referenc e**
aggR	5’GTATACACAAAAGAAGGAAGC3’	254	[1]
	5’ACAGAATCGTCAGCATCAGC3’		
lt	5’GCGACAAATTATACCGTGCT3’	708	[2]
	5’CCGAATTCTGTTATATATGT3’		
sta	5’CTGTATTGTCTTTTTCACCT3’	182	[2]
	5’GCACCCGGTACAAGCAGGAT3’		
eaeA	5’GACCCGGCACAAGCATAAGC3’	384	[3]
	5’CCACCTGCAGCAACAAGAGG3’		
bfp	5’CAATGGTGCTTGCGCTTGCT3’	324	[2]
	5’GCCGCTTTATCCAACCTGGT3’		
ipaH	5’GCTGGAAAAACTCAGTGCCT3’	424	[2]
	5’CCAGTCCGTAAATTCATTCT3’		
afaB/afaC	5’GCTGGGCAGCAAACTGATAACTCTC3’	750	[4]
	5’CATCAAGCTCTTTGTTCGTCCGCCG3’		
stx1	5’ATAAATCGCCATTCGTTGACTAC3’	180	[3]
	5’AGAACGCCCACTGAGATCATC3’		
stx2	5’GGCACTGTCTGAAACTGCTCC3’	255	[3]
	5’TCGCCAGTTATCTGACATTCTG3’		

Supplementary Table 2 – pathogenic diarrheagenic *E. coli* genes used to assign pathotypes in the bioinformatic analyses

Pathotype	Pathotype designation criteria	Gene name	Gene description	Genbank Accession #
ETEC	Presence of *lt* (both subunits) and/or *sta* genes	*eltA*	Heat-labile enterotoxin subunit A	AAA24685
		eltB	Heat-labile enterotoxin subunit B	AAA98064
		sta	Heat-stabile enterotoxin	WP_001353651
EPEC	EPECt ("typical"): presence of both *eaeA* and *bfpA*; EPECa ("atypical"): presence of *eaeA* and absence of *bfpA*	*eaeA*	Enterocyte effacement-encoded intimin protein	AAC38392
	escN	*bfpA*	Bundle-forming pilus	BAA84838
EIEC	*E. coli* isolates with ipaH were designated as EIEC	*ipaH*	pINV plasmid-encoded type-III effector protein	NP_858212
EAEC	Isolates were defined as EAEC if *aggR*, *aaiC*, and/or *aatA* were present	*aggR*	Aggregative adherence transcriptional regulator	QKN22446
		aaiC	Secreted protein	LT719075
		aatA	Biofilm formation protein	SJK83517
DAEC	The afa, dra, and daa operons are made up of a series of structural proteins and adhesins; isolates were considered DAEC if their draft genome sequences contained >50% of structural genes for at least one operon and 1 or more adhesin genes (*afaE, draE*, or F1845)			
		afaF-III	afa operon transcriptional regulator	CAA54112
		afaA	afa operon transcriptional regulator	CAW30797
		afaB-I	Afa operon periplasmic chaperone	CAW30798
		afaC-I	Afa operon anchoring protein variant	CAW30799
		afaC-III	Afa operon anchoring protein variant	CAA54117
		afaD	Afibrial adhesin subunit D	CAW30800
		afaE-I	Afimbrial adhesin subunit E variant	CAW30801
		afaE-II	Afimbrial adhesin subunit E variant	CAA59767
		afaE-III	Afimbrial adhesin subunit E variant	CAA54121
		afaE-V	Afimbrial adhesin subunit E variant	CAA62863
		draA	Dra operon transcriptional regulator	AAK16475
		draB	Dra operon periplasmic chaperone	AAK16476
		draC	Dra operon anchoring protein	AAK16477
		draD	Dra operon afibrial adhesin subunit D	AAK16478
		draP	Dra operon fimbriae coding cluster protein	AAK16479
		draE	Dra operon fimbrial adhesin subunit E variant	AAK16480
		draE-II	Dra operon fimbrial adhesin subunit E variant	AAB65153
		daaF	Daa operon transcriptional regulator	AAA23662
		daaA	Daa operon transcriptional regulator	AAA23663
		daaC	Daa operon anchoring protiein	ABU51870
		daaD	Daa Fimbrial adhesin subunit D	AAG10405
		F1845	Daa operon fimbrial adhesin	AAA23661
EHEC	Isolates that had stx and eaeA were designated as EHEC	*stx1 A*	Shiga-like toxin, subunits A and B	WP_000691354
		stx1 B	Shiga-like toxin, subunits A and B	WP_000752026
		eaeA	Locus of enterocyte effacement-encoded intimin protein	AAC38392

Supplementary Table 3 - *E. coli* isolate PCR, isolate WGS, and metagenome sequence pathotype identification, and qPCR results in copies/ng DNA for the 35 samples used for full comparisons.

Pathogen genes were considered detected when sequencing depth was above 0.1 for short read mapping. If at least half of the pathogenic genes of a single type were identified but coverages for these were below 0.1, or if less than half were below 0.1 with some reads at 0.1 and above (mixed), the pathogen was considered detected but low coverage and have a “LC” superscript maker (see table S4 for numbers, 39). Single genes with coverages less than 0.1 for any pathotype were confirmed with recruitment plots and calls were made based on coverage. For DAEC detection in the metagenome, 3 or more genes were required to be recovered to be considered “detected”. In the isolate, DAEC was considered detected if there were more than 3 genes recovered at an average depth of 0.1 or above. qPCR metrics for each pathotype tested per sample are also shown where available. Cells with “NT” indicate the marker was not tested for, “ND” indicates the marker was not detected via qPCR.

Pathotype analysis results								qPCR results (copies/ng DNA)				
***E. coli* isolate ID**	***MGID***	**Isolate pathotype PCR result**	**Pathotype of pathogen genes mapped to isolate assembly**	**Pathotype of isolate short reads mapped to pathogen genes**	**Pathotype of metagenome short reads mapped to pathogen genes**	**Isolate coverage in metagenome (TAD80 99% ANIr)**	**Relative abundance of isolate in metagenome**	**DAEC**	**ETEC_lt**	**ETEC_sta**	**EPEC_eae**	**EPEC_bfpA**
B001_5	MG_1	DAEC	DAEC	DAEC	DAEC/EAEC/ETEC	2.19	4.13%	NT	NT	NT	NT	NT
B109_1	MG_10	EAEC/ETEC	EAEC/ETEC	EAEC/ETEC	EAEC/ETEC	0.07	0.05%	NT	712.1	NT	NT	NT
B200_2	MG_15	ETEC	ETEC	ETEC	ETEC/EIEC	6.75	1.32%	NT	115.9	NT	NT	NT
B228_2	MG_14	EPECa	rpoB only	rpoB only	EPECa	0.07	0.06%	NT	NT	NT	NT	NT
B24_1	MG_5	DAEC	DAEC	DAEC	EIEC	0.01	0.03%	8.3	NT	8.3	NT	NT
B244_3	MG_12	ETEC	ETEC	ETEC	ETEC/EPEC	3.69	1.24%	NT	NT	NT	NT	NT
B255_1	MG_11	ETEC	ETEC	ETEC	ETEC/EAEC	2.11	0.54%	NT	NT	NT	NT	NT
B274_2	MG_13	DAEC	DAEC	DAEC	DAEC/EAEC	9.49	3.24%	NT	NT	NT	NT	NT
B295_2	MG_16	ETEC	ETEC	ETEC	ETEC/EAEC	10.83	3.25%	3.5	15112	14035.4	NT	NT
B45_2	MG_6	ETEC	ETEC	ETEC	ETEC/DAEC[LC]	17.89	3.72%	ND	41860.5	38500.7	NT	NT
B62_5	MG_3	ETEC	ETEC	ETEC	ETEC/EAEC	4.8	1.3	NT	NT	NT	NT	NT
B68_1	MG_4	ETEC	ETEC	ETEC	ETEC	1.46	0.46%	NT	1409.8	NT	NT	NT
B69_1	MG_8	EPECa	rpoB only	EPECa	EPECa/EAEC	3.47	1.09%	NT	NT	NT	NT	NT
B89_1	MG_9	DAEC	DAEC	DAEC	DAEC[LC] /EIEC	0.74	2.19%	44.9	NT	1.7	NT	NT
E124_6	MG_23	DAEC	DAEC	DAEC	DAEC/EAEC	3.26	0.98%	NT	NT	NT	NT	NT
E158	MG_24	DAEC	DAEC	DAEC	DAEC	106.03	35.61	97993.4	67.17	24.75	NT	NT
E162	MG_22	EPECa	EPECa	EPECa	EPECa	2.51	1.48%	NT	4.2	37.3	370.5	6.9
E184_3	MG_19	ETEC	rpoB only	EAEC	DAEC[LC]	28.25	10.69%	NT	NT	NT	NT	NT
E187_2	MG_21	EPECt	EPECt	EPECt	EPECt / DAEC	14.89	4.81%	1336.2	NT	1.3	3485.2	10990
E205	MG_20	EPECa	rpoB only	rpoB only	DAEC	0.62	0.31%	NT	NT	NT	NT	NT
E230_4	MG_25	DAEC	DAEC	DAEC	DAEC/EAEC	66.23	20.16%	1181.5	ND	1.6	NT	NT
E26	MG_18	DAEC	DAEC	DAEC	EPECa	0.11	0.12%	120.6	28.5	0.5	682.3	NT
E27	MG_17	DAEC	DAEC	DAEC	DAEC	7.83	2.49%	3886.8	NT	8.1	NT	NT
Q196	MG_32	DAEC	DAEC	DAEC	DAEC/EAEC	6.54	17.21%	8432.1	ND	ND	NT	NT
Q233	MG_35	EPECa	EPECa	EPECa	EPECa	0.25	0.10%	NT	NT	NT	60	NT
Q294	MG_33	ETEC	ETEC	ETEC	ETEC/ DAEC/EAEC/	64.43	10.72%	NT	59703.7	ND	NT	NT
Q300	MG_36	EPECa	NONE	EPECa/EIEC	EPECa	0	0.02%	NT	NT	NT	NT	NT
Q308	MG_26	DAEC	DAEC	DAEC	DAEC	0.81	0.22%	928.3	NT	0.6	NT	NT
Q310	MG_27	DAEC	DAEC	DAEC	DAEC	1.27	0.53%	NT	NT	NT	NT	NT
Q51	MG_31	DAEC	DAEC	DAEC	DAEC/EIEC/ETEC/EPECa	11.35	31.98%	5357.7	NT	NT	NT	NT
Q53	MG_34	ETEC	ETEC	ETEC/EPEC	ETEC/EAEC/EIEC	1.35	1.04%	NT	NT	NT	NT	NT
Q56	MG_29	DAEC	DAEC	DAEC	DAEC	77.08	25.16%	42649.5	0	10.6	NT	NT
Q65	MG_30	DAEC	DAEC	DAEC	DAEC	35.61	21.46%	64355.1	0	4.8	NT	NT
Q71_1	MG_55	ETEC	ETEC	ETEC	rpoB only	0.14	0.07%	NT	NT	NT	NT	NT
R126_2	MG_57	EPECa	NONE	rpoB only	EIEC	1.13	8.14%	NT	NT	NT	NT	NT

Supplementary Table 4—Read coverages of pathogenic genes from isolates and metagenomes

Comparison of pathogenic gene recovery at the read level of isolate and metagenome pairs. Trimmed short read sequences of isolate WGS and metagenomic shotgun sequencing were blasted against the pathogen gene reference sequence files using Magic Blast, then filtered using custom Python scripts (see methods). All recovered genes are represented in the table at untruncated depth, with "-" indicating no recovery at the read level. Read detection was done using same criteria as described in Table S2. In one case, isolate B68_1 had a single DAEC gene recovered at above a depth of 0.1 but this was not considered as "detected" since more than 3 genes were required. This, and other DAEC cases where the detection criteria were not met, are marked with asterisks. See Table S2 for details on pathogen genes.

**Isolate read coverage of pathogen genes**							**Metagenome read coverage of pathogen genes**						
***E. coli* isolate ID**	**DAEC**	**EAEC**	**EPEC**	**ETEC**	**EIEC**	**rpoB**	***Metagenome ID***	**DAEC**	**EAEC**	**EPEC**	**ETEC**	**EIEC**	**rpoB**
B001_5	**18.78**	-	-	-	-	32.46	MG_1	**0.96**	**1.09**	-	**0.31**	-	8.45
B109_1	-	**6.39**	-	**6.50**	-	18.83	MG_10	-	**0.14**	-	**0.77**	-	0.45
B200_2	-	-	-	**5.13**	-	23.89	MG_15	-	-	-	**0.18**	**7.98**	15.88
B228_2	-	-	-	-	-	30.24	MG_14	-	-	**0.21**	-	-	0.61
B24_1	**5.58**	-	-	-	-	37.04	MG_5	-	-	-	-	**0.28**	0.29
B244_3	-	-	-	**14.05**	-	29.80	MG_12	0.06*	-	**0.61**	**6.31**	-	9.47
B255_1	-	-	-	**7.61**	-	7.61	MG_11	0.04*	**3.44**	-	**7.90**	-	6.72
B274_2	**0.89**	-	-	-	-	19.14	MG_13	**3.41**	**0.65**	-	-	-	17.26
B295_2	-	-	**9.99**	-	-	29.32	MG_16	-	**1.43**	-	**19.41**	-	18.91
B45_2	0.07	-	-	**26.72**	-	17.01	MG_6	**0.09**	-	-	**43.95**	-	26.66
B62_5	-	-	-	**9.01**	-	21.73	MG_3	**6.72**	-	-	**11.68**	-	8.75
B68_1	0.31*	-	-	**3.77**	-	46.90	MG_4	-	-	-	**1.27**	-	3.18
B69_1	-	-	**0.10**	-	-	36.27	MG_8	**0.29**	**1.50**	**0.23**	-	-	5.65
B89_1	**5.45**	-	-	-	-	33.17	MG_9	**0.08**	-	-	-	**0.61**	2.94
E124_6	**4.05**	-	-	-	-	22.77	MG_23	**0.38**	**5.84**	-	-	-	23.06
E158	**14.94**	-	-	-	-	33.54	MG_24	**41.22**	-	-	-	-	162.49
E162	-	-	**11.80**	-	-	26.12	MG_22	-	-	**0.61**	-	-	20.44
E184_3	-	**0.46**	-	-	-	27.15	MG_19	**0.05**	-	-	-	-	45.39
E187_2	-	-	**15.15**	-	-	18.32	MG_21	**0.67**	-	**7.97**	-	-	142.06
E205	-	-	-	-	-	15.34	MG_20	**0.52**	-	-	-	-	1.84
E230_4	**1.11**	-	-	-	-	23.24	MG_25	**1.00**	**2.23**	-	-	-	123.11
E26	**7.18**	-	-	-	-	24.40	MG_18	0.06*	-	**0.21**	-	-	0.69
E27	**6.48**	-	-	-	-	16.74	MG_17	**2.23**	-	-	-	-	13.98
Q196	**5.43**	-	-	-	-	23.47	MG_32	**2.58**	**0.99**	-	-	-	28.01
Q233	-	-	**5.40**	-	-	10.85	MG_35	-	-	**0.24**	-	-	1.00
Q294	-	-	-	**40.29**	-	32.05	MG_33	**1.70**	**5.68**	-	**72.39**	-	98.90
Q300	0.08	-	**0.12**	-	**0.62**	30.67	MG_36	-	-	**0.20**	-	-	0.05
Q308	**8.86**	-	0.08	-	-	38.30	MG_26	**0.41**	-	-	-	-	1.74
Q310	**12.75**	-	-	-	-	30.30	MG_27	**1.55**	-	-	-	-	3.08
Q51	**6.15**	-	-	-	-	22.11	MG_31	**3.78**	-	-	**0.60**	**6.09**	39.00
Q53	-	-	**0.25**	**10.43**	-	24.68	MG_34	-	**0.58**	-	**3.51**	**6.62**	4.99
Q56	**7.01**	-	-	-	-	24.71	MG_29	**31.45**	-	-	-	-	103.43
Q65	**5.26**	-	-	-	-	43.01	MG_30	**16.28**	-	-	-	-	57.97
Q71_1	-	-	-	**5.25**	-	11.46	MG_55	-	-	-	-	-	0.36
R126_2	-	-	-	-	-	13.01	MG_57	-	-	-	-	**32.57**	7.47

Supplementary Table 5 - Results of primer and DEC diagnostic virulence gene mapping against isolate contigs.

Pathogen genes were mapped against isolate contigs, and contigs with positive matches to genes were blasted against the non-redundant (NR) database of NCBI using BLASTn. Generally, both primers and pathogen genes mapped to the same contigs, though alternate mappings were common (not shown). The “location of both” column indicates whether the main contig matches in common between primer and pathogen gene mappings were to *E. coli* chromosomes (“CHROM”), plasmids (“PLAS”), a mix of both (“MIX”) or if no mappings of primers or pathogen genes were recorded (“NA”). The first NCBI accession number for the represented contig is shown in the NCBI accession column, though there were generally numerous results to different plasmid and chromosome loci.

E. coli isolate ID	*Primer blast call*	*Pathogen gene blast call*	*Location of both*	*NCBI accession*	*Notes*
B001_5	DAEC	DAEC	CHROM	CP050202.1	
B24_1	DAEC	DAEC	CHROM	CP077379.1	
B89_1	DAEC	DAEC	CHROM	CP034404.1	
E124_6	DAEC	DAEC	CHROM	CP038299.1	
E158	DAEC	DAEC	CHROM	CP113493.1	
E230	DAEC/ETEC	DAEC	CHROM	CP113493.1	
Q196	DAEC	DAEC	CHROM	CP061339.1	
Q65	DAEC	DAEC	CHROM	CP077379.1	
Q310	DAEC/ETEC	DAEC	CHROM	CP088725.1	
E162	EPEC/ETEC	EPEC	CHROM	CP038394.1	
E187_2	EPEC	EPEC	CHROM	CP042948.1	
Q233	EPEC/DAEC	EPEC	CHROM	CP010238.1	
B62_5	ETEC	ETEC	PLAS	CP024277.1	
B200	ETEC	ETEC	PLAS	CP024669.1	
B244_3	ETEC	ETEC	PLAS	CP024277.1	
B295_2	ETEC	ETEC	PLAS	CP024277.1	
B45	ETEC	ETEC	PLAS	CP024277.1	
B68_1	NONE/LC DAEC	ETEC	PLAS	CP025912.1	both plasmids despite low primer mapping
Q294	ETEC	ETEC	PLAS	CP024277.1	
Q53	ETEC	ETEC	PLAS	CP029980.1	
Q71	ETEC	ETEC	PLAS	CP024259.1	
B109_1	ETEC	ETEC/EAEC	PLAS	CP022280.1	lower coverages of both (76% cov) all plasmids
B274_2	DAEC	DAEC	MIX	NA	contig in common is chrom, primers map to DAEC plasmid too
E26	DAEC	DAEC	MIX	NA	contig in common is chrom, primers map to DAEC plasmid too
E27	DAEC	DAEC	MIX	NA	contig in common is chrom, primers map to DAEC plasmid too
Q308	DAEC	DAEC	MIX	NA	contig in common is chrom, primers map to DAEC plasmid too
Q51	DAEC	DAEC	MIX	NA	contig in common is chrom, primers map to DAEC plasmid too
Q56	DAEC	DAEC	MIX	NA	contig in common is chrom, primers map to DAEC plasmid too
B255_1	ETEC	ETEC	MIX	NA	primer and pathgene share contigs, but primer maps to another contig which gives a plasmid result (pathgenes are all chromosome)
E184_3	none	EAEC	NA	NA	pathogen genes map to CHROM
B69_1	none	EPEC	NA	NA	pathogen genes map to CHROM
Q300	none	rpoB only	NA	NA	
E205	none	rpoB only	NA	NA	
R126_2	none	rpoB only	NA	NA	
B228_2	none	rpoB only	NA	NA	rpob maps to CHROM

Supplementary Table 6: Comparison of isolate genomes and MAGs in the 13 samples with both diarrheal *E. coli* isolates and high-quality *E. coli* MAGs. Isolate pathotype, metagenome pathotype based on read mapping, and MAG pathotype provided for comparison. ANI distances between MAG and isolate indicate relatedness between MAG-isolate pairs (% ANI). Pathogen gene coverages in the metagenome and isolate are also provided for comparison. The *rpoB* clonality section shows relatedness based on *rpoB* of MAGs and isolates. 100% *rpoB* ANI between MAG an isolate indicates fully clonal pairs. The “CRR” labels in the final two columns stands for “competitive read recruitment” and indicates the coverages of MAG-isolate pairs in the metagenome from a competitive read recruitment assay.

Summary of pathotype analysis and abundance between MAG and isolate										Summary of rpoB clonality and abundance between MAG and isolate				
isolate ID	*MGID*	Isolate pathotype	Metagenome pathotype	MAG type	ANI between MAG and ISO	pathgene coverage in isolate	pathgene coverage in MG	Isolate coverage in metagenome (TAD80 > 99% ANIr)	MAG coverage in metagenome (TAD80 >99% ANIr)	rpob coverage in isolate	rpob coverage in metagenome	rpoB ANI between MAG and ISO	CRR MG to ISO rpob (TAD80 99% ANIr)	CRR MG to MAG rpob (TAD80 99% ANIr)
B200_2	MG_15	ETEC	ETEC/EIEC	NONE	98.64	5.13	0.18	6.75	9.37	23.89	15.88	99.81	7.42	15.03
B295_2	MG_16	ETEC	ETEC/EAEC	NONE	99.63	6.51	19.41	10.83	10.85	25.56	18.91	100.00	NA	NA
B45_2	MG_6	ETEC	ETEC/DAEC^{LC}	NONE	99.93	26.72	43.95	17.89	17.29	17.01	26.66	100.00	NA	NA
B62_5	MG_3	ETEC	ETEC/EAEC	NONE	99.09	9.01	11.69	4.8	5.59	21.73	8.75	100.00	NA	NA
E124_6	MG_23	DAEC	DAEC/EAEC	NONE	96.84	4.05	0.38	3.26	15.12	22.77	23.06	100.00	NA	NA
E158	MG_24	DAEC	DAEC	DAEC	99.54	15.00	43.59	106.03	99.78	32.48	164.04	100.00	NA	NA
E184_3	MG_19	EAEC	DAEC^{LC}	NONE	99.60	0.46	0.05	28.25	25.67	27.15	45.96	100.00	NA	NA
E230_4	MG_25	DAEC	DAEC/EAEC	NONE	99.29	1.11	1.00	66.23	86.19	23.24	123.11	99.91	57.38	65.44
Q196	MG_32	DAEC	DAEC/EAEC	DAEC	97.16	5.43	2.58	6.54	19.64	23.47	28.01	99.45	8.95	18.33
Q294	MG_33	ETEC	ETEC/ DAEC/EAEC/	NONE	99.69	40.30	72.39	64.43	64.05	49.01	98.90	100.00	NA	NA
Q51	MG_31	DAEC	DAEC/EIEC/ETEC/EPECa	DAEC	96.86	6.15	3.78	11.35	15.72	22.11	39.00	99.16	11.45	24.35
Q56	MG_29	DAEC	DAEC	DAEC	99.97	7.01	31.46	77.08	75.74	24.71	103.43	100.00	NA	NA
Q65	MG_30	DAEC	DAEC	DAEC	99.17	5.26	16.28	35.61	44.45	43.01	57.97	99.95	27.88	30.28

Supplementary table 7 - Primers and annealing temperatures used for qPCR assays and their source references.

Target (gene)	Primer name	Primer sequence (5'-3')	Annealing temperature (ºC)	Reference
Total bacteria (16S rRNA)	U16SRT-F	ACTCCTACGGGAGGCAGCAGT	61.5	Clifford et al. 2012
	U16SRT-R	TATTACCGCGGCTGCTGGC		
DAEC (*daaC*)	daaC-F	ATTACGTCATCCGGGAAGCACACA	60	Souza et al. 2013
	daaC-R	GCTTGCTCATAAAGCCGCAGACAA		
ETEC (*lt*)	lt-F	GGCAGGCAAAAGAGAAATGG	60	Lithigius et al. 2008
	lt-R	TCCTTCATCCTTTCAATGGCT		
ETEC (*sta*)	sta-F	ACCTTTCGCTCAGGATGCTAAACC	60	Youmans et al. 2014
	sta-R	AATAGCACCCGGTACAAGCAGGAT		
Inhibition control assay	ICA-F	CTAACCTTCGTGATGAGCAATCG	60	Deer et al. 2010
	ICA-R	GATCAGCTACGTGAGGTCCTAC		

Supplementary table 8 - Summary of qPCR assay performance.

Target (gene)	Slope	R^2	y-intercept	Efficiency (%)
Total bacteria (16S rRNA)	-3.67	0.996	42.13	87.5
DAEC (*daaC*)	-3.408	0.995	39.22	97
ETEC (*lt*)	-3.42	0.985	39.45	96
ETEC (*sta*)	-3.25	0.984	39.4	103.2
Inhibition control assay	-3.35	0.991	38.8	98.8

Supplementary Table 9 – Results of pathogen gene blasts against unbinned metagenome contigs

Pathogen genes were mapped against unbinned metagenome contigs, and contigs with positive matches to genes were blasted against the non-redundant (NR) database of NCBI using BLASTn. Results from each contig that returned a result are shown. Metagenome contigs with no recovered pathogen genes are not included. Contig pathotypes as well as their resulting matches to either *E. coli* chromosomes (“CHROM”), plasmids (“PLAS”) or a mix of both (“MIX”) are shown. One sample’s *rpoB* gene did not return a blast result (MG_24). One metagenome had mixed results with *E. coli* and *Shigella*

sp., with only results from *E. coli* shown (MG_31, marked with asterisk). One metagenome (MG_57) returned all results to *Shigella* sp (not shown).

Metagenome ID	*Metagenome pathotype based on short read mapping to pathogen genes*	*Contig pathotype*	*Contig location in E. coli genome*	*NCBI Accession*
**MG_1**	DAEC/EAEC/ETEC	DAEC	CHROM	CP033762.1
	DAEC/EAEC/ETEC	DAEC	CHROM	CP050202.1
**MG_11**	ETEC/EAEC	EAEC	PLASMID	CP024249.1
	ETEC/EAEC	ETEC	PLASMID	CP035868.1
	ETEC/EAEC	ETEC	PLASMID	CP024249.1
**MG_12**	ETEC/EPEC	ETEC	PLASMID	CP024277.1
**MG_13**	DAEC/EAEC	rpob	CHROM	CP093368.1
	DAEC/EAEC	DAEC	PLASMID	CP091692.1
**MG_15**	ETEC/EIEC	rpob	CHROM	CP024978.1
	ETEC/EIEC	EIEC	CHROM	CP050865.1
**MG_16**	ETEC/EAEC	rpob	CHROM	CP013190.1
	ETEC/EAEC	ETEC	PLASMID	CP024277.1
**MG_17**	DAEC	rpob	CHROM	CP124515.1
	DAEC	DAEC	CHROM	CP015159.1
	DAEC	DAEC	CHROM	CP095137.1
	DAEC	DAEC	CHROM	CP015159.1
	DAEC	DAEC	CHROM	CP015159.1
**MG_19**	$DAEC^{LC}$	rpob	CHROM	CP035836.1
**MG_21**	EPECt / DAEC	EPEC	PLASMID	AP014804.1
**MG_23**	DAEC/EAEC	rpob	CHROM	CP041678.1
	DAEC/EAEC	EAEC	PLASMID	CP056903.1
**MG_24**	DAEC	rpob	NO RESULT	NO RESULT
	DAEC	DAEC	CHROM	CP015076.1
	DAEC	DAEC	CHROM	CP113493.1
	DAEC	DAEC	CHROM	CP113493.1
	DAEC	DAEC	CHROM	CP054828.1
**MG_25**	DAEC/EAEC	rpob	CHROM	CP041678.1
	DAEC/EAEC	DAEC	CHROM	CP088779.1
**MG_27**	DAEC	DAEC	CHROM	CP048337.1
	DAEC	DAEC	CHROM	CP088725.1
	DAEC	DAEC	CHROM	CP048337.1
	DAEC	DAEC	CHROM	CP088725.1
**MG_29**	DAEC	rpob	CHROM	AP022351.1
	DAEC	DAEC	CHROM	CP015159.1
**MG_3**	ETEC/EAEC	rpob	CHROM	CP123951.1
	ETEC/EAEC	EAEC	PLASMID	CP024249.1
	ETEC/EAEC	ETEC	PLASMID	CP023350.1
**MG_30**	DAEC	rpob	CHROM	CP024978.1
	DAEC	DAEC	CHROM	CP077379.1
	DAEC	DAEC	CHROM	CP077379.1
**MG_31***	DAEC/EIEC/ETEC/EPECa	DAEC	CHROM	CP095137.1
	DAEC/EIEC/ETEC/EPECa	rpob	CHROM	CP014197.1
	DAEC/EIEC/ETEC/EPECa	EIEC	PLASMID	CP001064.1
**MG_32**	DAEC/EAEC	rpob	CHROM	CP041678.1
	DAEC/EAEC	DAEC	CHROM	CP076693.1
	DAEC/EAEC	DAEC	CHROM	CP048337.1
	DAEC/EAEC	DAEC	CHROM	CP054317.1
**MG_33**	ETEC/ DAEC/EAEC/	DAEC	CHROM	CP015159.1
	ETEC/ DAEC/EAEC/	EAEC	PLASMID	CP091392.1
	ETEC/ DAEC/EAEC/	EAEC	MIX	CP022280.1
	ETEC/ DAEC/EAEC/	ETEC	PLASMID	CP029980.1
	ETEC/ DAEC/EAEC/	DAEC	CHROM	CP015159.1
	ETEC/ DAEC/EAEC/	DAEC	PLASMID	AP022816.1
	ETEC/ DAEC/EAEC/	DAEC	CHROM	CP095137.1
	ETEC/ DAEC/EAEC/	DAEC	CHROM	CP054317.1
	ETEC/ DAEC/EAEC/	DAEC	CHROM	CP015159.1
	ETEC/ DAEC/EAEC/	DAEC	CHROM	CP015159.1
**MG_34**	ETEC/EAEC/EIEC	EIEC	PLASMID	CP053752.1
	ETEC/EAEC/EIEC	ETEC	PLASMID	CP029980.1
**MG_6**	$ETEC/DAEC^{LC}$	rpob	CHROM	AP023235.1
	$ETEC/DAEC^{LC}$	ETEC	PLASMID	CP024277.1
**MG_8**	EPECa/EAEC	rpob	CHROM	CP123963.1

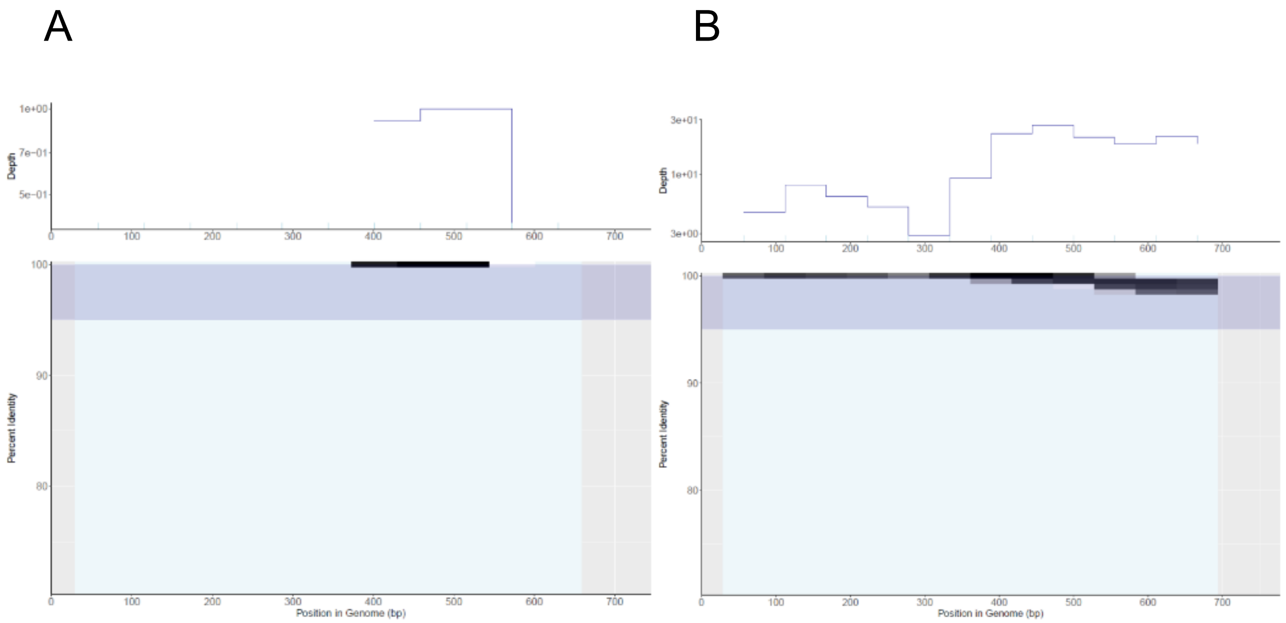

Supplementary Figure 1 - Recruitment plots of isolate short reads mapped to DEC diagnostic virulence genes from isolate sample R119_3. The top line plot represents sequencing depth across the reference gene, indicating how many reads recruit to each base pair position in log scale. Lower blue panels represent reads recruited by location in genome (x-axis) vs read identity greater than 95% (darker blue panel overlaid on light blue panel, y-axis) or lower than 95% identity (in light blue). **A)** Example of a low abundance call for read recruitment, one read recruited to *afab-I* sequence (DAEC) with a coverage of 0.24. **B)** Example of high abundance read recruitment to *eltA* sequence (ETEC) with 3-8 reads recruiting at various points along the gene sequence and coverage of 11.97. In this case, isolate R119_3 was considered negative (not detectable) for DAEC since only a single gene was recovered and was considered positive for ETEC.

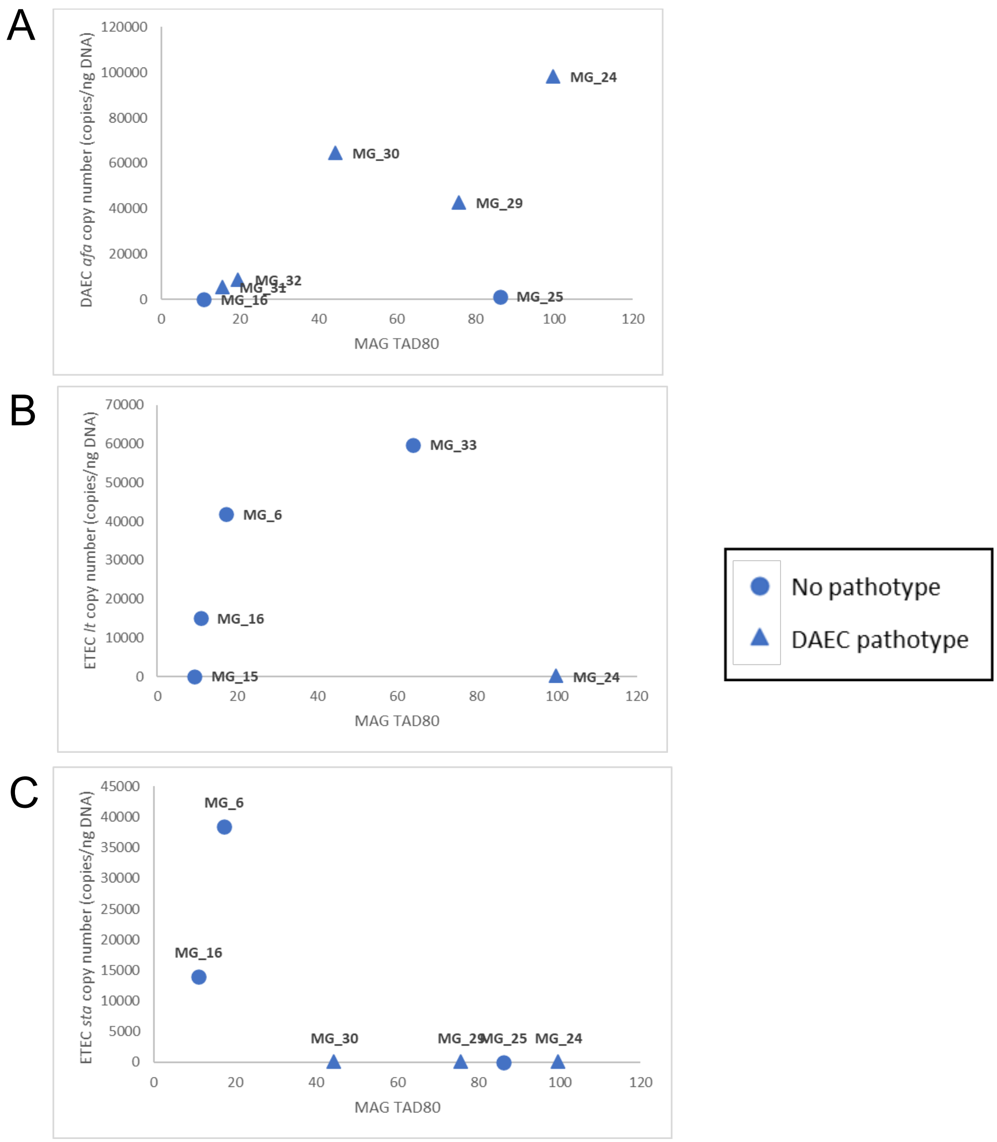

Supplementary Figure 2 - Dot plots representing relationships between MAG coverages in the metagenomes as TAD80 vs qPCR copy numbers for DAEC-associated *afa* genes **(A)** and ETEC-diagnostic gese *lt* **(B)** and *sta* **(C)** for the 13 samples for which we had both qPCR results and high-quality *E. coli* MAGs. Circles represent MAGs for which no pathotype was identified in the assembly, and triangles represent the MAGs where a DAEC pathotype was determined. All DAEC-pathotype MAGs were represented and there was a notable trend between increasing MAG TAD80 and increasing *afa* copy number (A). For ETEC *lt*, there was a small increase in the *lt* copy numbers as TAD80 of the MAGs in the metagenomes increased (B), but same trend was not observed for ETEC *sta* genes (C). The no-pathotype MAGs were all extracted from samples with ETEC pathotype designation, but none of these MAGs contained ETEC genes.
